# Emotion Understanding Correlates With Parental Emotional Expressivity in Chinese Youths With Hearing Loss and Typical Hearing

**DOI:** 10.3389/fpsyg.2021.662356

**Published:** 2021-06-29

**Authors:** Yousong Hu, Shuyang Dong, Fang Guan, Outong Chen, Jun Chen, Shurong Xu

**Affiliations:** ^1^School of Psychology, South China Normal University, Guangzhou, China; ^2^Key Laboratory of Brain, Cognition and Education Sciences (South China Normal University), Ministry of Education, Guangzhou, China; ^3^Center for Studies of Psychological Application South China Normal University, Guangzhou, China; ^4^Guangdong Key Laboratory of Mental Health and Cognitive Science, South China Normal University, Guangzhou, China; ^5^Department of Developmental Psychology, Utrecht University, Utrecht, Netherlands; ^6^Department of Psychology, Tsinghua University, Beijing, China; ^7^Department of Normal College and School of Teacher Education, Qingdao University, Qingdao, China; ^8^Luoding Middle School, Luo Ding, China

**Keywords:** emotion understanding, negative emotional expressivity, positive emotional expressivity, hearing loss, typical hearing

## Abstract

This study aimed at examining the differences between Chinese youths with hearing loss (HL) and with typical hearing (TH) in emotion understanding (EU), parental emotional expressivity, and the associations between EU and parental emotional expressivity. The participants were 282 youths with HL (14.58 ± 3.42 years old) and 350 youths with TH (11.69 ± 2.49 years old). EU was measured by four visual-mode tasks, of which two involve language comprehension while the others do not. Parents reported positive and negative emotional expressivity on the Self-Expressiveness in the Family Questionnaire. Covariates were controlled for including socioeconomic status, parent gender, youth gender, age, intelligence, and teacher-reported comprehension difficulties. Results showed that the four EU tasks were more challenging for the youths with HL than for the youths with TH. The interaction effect of the two groups × 4 tasks was not significant, suggesting that the differences between the two groups of youths in EU were generally similar across the four tasks. The parents of the youths with HL did not differ from the parents of the youths with TH in how often they displayed positive and negative emotional expressivity. Multigroup regression analyses revealed that negative emotional expressivity was negatively related to EU in the youths with HL but not in the youths with TH. However, these two regression coefficients were not significantly different. Positive emotional expressivity was not related to EU in either group. In conclusion, this study extends the knowledge about the EU of Chinese youths with HL and emotion-related socialization of the parents of these youths.

## Introduction

Emotion understanding (EU) refers to the knowledge of one's own and others' emotions, such as recognizing and differentiating facial expressions of different emotions (Pons et al., 2004). Studies of children and adolescents with typical development commonly focus on the understanding of four emotions [i.e., happiness, sadness, anger, and fear (McClure, 2000)]. From middle childhood onward, typically developing children gradually refine their multifaceted knowledge of these four emotions [e.g., auditory cues of emotions or emotional cues in facial expressions (Pons et al., 2003)], and, by adolescence, most individuals can already classify these four emotions accurately (McClure, 2000). However, whether this is the same case for school-aged children and adolescents with hearing loss (HL) needs to be examined. The youths with HL are commonly born to hearing parents or live in a predominantly hearing environment, but they have the developmental experience that is quite different from that of their peers with typical hearing (TH), which might influence their EU development. Particularly, thus far, little do we know about the EU of the youths with HL from non-Western cultures (e.g., the Chinese culture), even for the understanding of the four emotions that have been most commonly examined among the youths with TH. Therefore, the first aim of the current study was to address this research gap by examining between- and within-group differences across various EU tasks with a large sample of Chinese youths with HL and with TH. Moreover, to some extent, individual differences in EU have been related to parental emotional expressivity, a style of expressing emotions through verbal or non-verbal behaviors (see Ogren and Johnson, 2020 for a review). Yet, it remains unclear whether the parents of youths with HL differ from the parents of youths with TH in how often they express positive and negative emotions at home and whether the associations between EU and these two aspects of parental emotional expressivity are similar between the two groups of youths. Therefore, the second aim was to address these research questions.

### Group Differences in Emotion Understanding

With respect to the between-group difference in EU, of importance is to differentiate between the tasks conducted through different sensory modes (i.e., visual or auditory). The youths with HL have limited access to auditory cues when processing emotions, which interferes with the integration of information and reduces the accuracy of evaluating emotional signals (Most and Aviner, 2009). Compared with the youths with TH, the youths with HL have lower accuracy in the auditory-mode EU tasks (Most et al., 1993; Most and Aviner, 2009). In contrast, the results are less consistent for the between-group difference in the EU tasks administered through the visual mode. While some studies found that EU in the visual mode is resilient to the effects of HL, thus showing a similar performance between the youths with HL and the youths with TH (Hosie et al., 1998; Hopyan-Misakyan et al., 2009; Most and Aviner, 2009); other studies found that the EU of the youths with HL is less optimal than that of the youths with TH (Most et al., 1993; Dyck and Denver, 2003).

Such inconsistency in the visual-mode EU tasks may be explained, at least in part, by the differences among these tasks in the requirements of language skills. Reduced access to auditory cues has profound influences on the development of language skills. In theory, the emergence of a less optimal EU in the youths with HL may be a product of delayed language development, such as having comprehension difficulties, potentially due to the linguistic deprivation in these youths (Dyck et al., 2004; Sidera et al., 2016). Correspondingly, to further understand the inconsistency in the literature on the between-group differences in EU, it is important to dive into the specific paradigms and identify the differences between these paradigms in, for example, involvement of language comprehension.

The paradigms in which language comprehension is involved include (1) matching emotion words with facial expressions and (2) identifying (causes of) emotions in stories or descriptive contexts. By contrast, language comprehension is less involved in the other paradigms, such as (3) recognizing and matching static facial expressions and (4) identifying facial expressions in dynamic scenes. For the first two paradigms, the results in the literature have been consistent, showing that compared with the youths with TH, the youths with HL had less knowledge of emotion words (Dyck and Denver, 2003; Dyck et al., 2004) and relatively delayed development in identifying emotions in stories or descriptive scenarios (Dyck and Denver, 2003; Dyck et al., 2004; Gray et al., 2007). In contrast, for the latter two paradigms, some studies found that compared with the youths with TH, the youths with HL had similar performance on recognizing and matching static facial expressions (Hosie et al., 1998; Hopyan-Misakyan et al., 2009) and identifying facial expressions in dynamic scenes (Most and Aviner, 2009; Jones et al., 2018), whereas others found that the youths with HL performed somewhat less optimally on these two paradigms (Most et al., 1993; Ludlow et al., 2010).

These findings suggest the possibility that, because of the potential within-group variations in different EU tasks, especially for the youths with HL, the between-group differences may vary across the two types of paradigms (i.e., whether or not language comprehension is involved). For one, the youths with HL may presumably obtain accuracy lower than their peers with TH on the EU tasks that involve language comprehension due to their delayed development of language skills. For another, whether or not the performance on the EU tasks that demand less on language comprehension would be different between the youths with HL and with TH is still unclear, and some other factors may contribute to the mixed results on these tasks. For instance, with the increase of ecological validity of stimuli, the group difference in EU seems to diminish between the two groups of youths (Ludlow et al., 2010; Jones et al., 2018), such that the youths with HL may be more likely to catch up with their peers with TH when human faces, instead of cartoon faces, are presented. Second, most of these studies have assessed child intelligence or/and language skills (e.g., Jones et al., 2018), but the potential influences of these factors have not been controlled when analyzing between-group differences in EU. The extent to which these factors confound with the group difference in EU may be different across studies, dependent on which assessment tools were used. Finally, small sample size and a related power issue should also be taken into account when interpreting these previous findings. To advance the knowledge of EU development, it is thus important to maximally account for these other factors and further explore the interaction effect between the groups and tasks to delineate possible variations in how large the mean-level differences might be between the youths with HL and with TH across various EU tasks.

### Emotion Understanding Among Chinese Youths With Hearing Loss

These aforementioned findings, however, are based exclusively on samples from Western cultures. There is evidence indicating that significant cultural differences exist in EU (Molina et al., 2014). Thus, of importance is to investigate such group differences in EU in youths from non-Western cultures (e.g., Chinese youths). With respect to Chinese youths, the previous studies have found a consistent between-group difference in EU, showing that compared with their peers with TH, the Chinese children with HL (Wang et al., 2019) and the Chinese adolescents with HL (Gu et al., 2019) had lower accuracy in the visual-mode EU tasks. Such results on the school-aged children and adolescents with HL (in comparison with their peers with TH) should be understood in consideration of the special education system in China.

In China, except for the very few who can satisfactorily complete the oral rehabilitation training in early childhood, almost all children and adolescents with HL have to complete 9-year compulsory education in special education schools (Lytle et al., 2005; Li and Prevatt, 2010). Many special education schools are residential, and they provide various education programs for children and adolescents with different disabilities (e.g., deafness or blindness) but separately in different classrooms. For the youths with HL, the special education program has its curriculum, which mainly focuses on assisting these youths in acquiring hearing and speech skills. In the meantime, these youths also need to take the same curriculum prepared for the youths without disabilities, for which the instructional methodology emphasizes skill and drill through memorization (Li and Prevatt, 2010; Wang and Andrews, 2017). Unfortunately, the current education system does not stress on improving social–emotional competencies of the youths with HL, and these youths have been experiencing high levels of fear and anxiety, owing to their disability (Li and Prevatt, 2010). Furthermore, the instructions typically used by teachers are not suitable for the acquisition of content knowledge and less effective in facilitating some language-based skills (e.g., expressive language) (Li and Prevatt, 2010; Wang and Andrews, 2017).

Generally speaking, the youths with HL already have fewer opportunities for incidental learning of knowledge of emotions, owing to their fewer interpersonal interactions with hearing individuals [indirect, unplanned, unstructured learning of emotions (Netten et al., 2015)]. Yet the current special education program in China does not provide coaching for the youths with HL to advance their knowledge of emotion either. As such, we surmise that the Chinese youths with HL might find all the EU tasks more challenging than their peers with TH. Specifically, the studies by Gu et al. (2019) and Wang et al. (2019) have demonstrated that compared with their peers with TH, the Chinese youths with HL, indeed, had less optimal performance on the tasks in which language comprehension was less involved. Moreover, because the youths with HL are more likely to encounter comprehension difficulties (Kyle and Cain, 2015), they are presumably to find the EU tasks that require the comprehension of emotion words or descriptive scenarios even more challenging. Correspondingly, it is possible that the Chinese youths with HL may differ more from the youths with TH on the EU tasks that involve language comprehension in comparison with the EU tasks that require less language comprehension.

The existing evidence, nonetheless, is still too scarce to draw a firm conclusion as the studies by Gu et al. (2019) and Wang et al. (2019) did not measure the EU tasks that involve language comprehension, did not include and examine both children and adolescents simultaneously, and the sample size of these two studies was quite small. To address these limitations, we drew from a large sample of the Chinese youths with HL and with TH to measure their EU in four visual-mode tasks (i.e., matching static facial expressions, identifying facial expressions in dynamic scenes, matching emotion words with facial expressions, and identifying emotions in descriptive contexts). We aimed at investigating the between-group difference in the overall performance on these EU tasks. In addition to this principal aim, if there is a significant between-group difference in EU, we also examined the interaction effect of groups and tasks to illustrate whether the between-group differences would be larger in the EU tasks in which language comprehension is critical.

### Parental Emotional Expressivity and Emotion Understanding

Furthermore, the group difference in EU has also come to be understood in light of the different amounts of emotion-related socialization. Theorists have proposed that the youths with HL lack sufficient opportunities to interact and communicate with parents (Rieffe and Terwogt, 2006), while these occasions are where EU might be acquired and calibrated. Parental emotional expressivity represents one of such opportunities. Parents express positive and negative emotions toward their children and the children could potentially pick up and learn the emotional cues in such typical expressions of emotions (Halberstadt and Eaton, 2002).

To our best knowledge, however, no studies have examined emotional expressivity in the parents of youths with HL. Due to a lack of research, two questions are of particular interest. First, do the parents of youths with HL differ from the parents of youths with TH in how often they express positive and negative emotions? Such differences are possible for negative emotional expressivity because the parents of youths with HL often experience communication difficulties with their children. Such experience was said to trigger parental stress and frustration (Knutson et al., 2004), the negative influence of which might further spillover to parent–child relationship, namely, parents may express negative emotions toward their children if they could not effectively regulate their own experience of these negative emotions (Hu et al., 2019). Specifically, the mothers of youths with HL have been found to be more authoritarian in their parenting and gave directives more often compared with the mothers of youths with TH (Knutson et al., 2004; Ekim and Ocakci, 2016). At least, for the Chinese parents, this authoritarian style of parenting is typically characterized as excessive expressions of negative emotions, and this parenting style was moderately associated with more negative emotional expressivity (Chen et al., 2011). However, thus far, it remains less clear whether the two groups of parents may differ in the frequency of expressing positive emotions. One study found that the parents of the youths with HL had relatively lower scores of warmth compared with the parents of youths with TH (Ekim and Ocakci, 2016), a parenting dimension that is somewhat relevant to the expressions of positive emotions. To sum up, it is possible that the parents of youths with HL may be more likely to display negative emotions compared with the parents of youths with TH. But there is a need to explore and examine whether these two groups of parents differ in how often they express positive emotions at home.

The second question is whether the associations between parental emotional expressivity and EU are similar for the youths with HL and the youths with TH. A meta-analytic study has shown that both positive and negative emotional expressivities are not significantly related to EU and the studies reviewed were generally similar in the strength of the associations (Halberstadt and Eaton, 2002). This indicates that the associations between parental emotional expressivity and EU may be generally weak and similar across different samples (although the studies reviewed did not examine the EU of the youths with HL). Indeed, the indirect evidence has supported this possibility, showing that the associations between positive as well as negative parenting and empathy are similar for the youths with HL and the youths with TH (Ketelaar et al., 2017). On the other hand, researchers have also argued that because the youths with HL may miss out on many interpersonal experiences with others (e.g., peers), their social–emotional development depends substantially on the socialization efforts provided by their parents (Ketelaar et al., 2017). This situation might be more salient for the Chinese youths with HL because, first, they are said to be overprotected by their parents (Li and Prevatt, 2010), thus having fewer opportunities to interact with the majority of the individuals outside school and home (e.g., peers with TH or strangers); and second, they have fewer peers with HL to interact within the classroom and are less likely to be allowed to interact with these peers after school. As such, the youths with HL may not have comparable numbers of interactions with peers, and, correspondingly, the interactions with parents take up a relatively high proportion of social interactions that these youths could have in everyday life. As a result, the EU of Chinese youths with HL may be more likely to depend on the influences of their parents. The strength of associations between parental emotional expressivity and EU may be stronger among these youths than among the youths with TH.

### The Present Study

In all, the aims of our research were 2-fold. First, we aimed at comparing the mean-level differences between the youths with HL and the youths with TH in the four EU tasks. We hypothesized that compared with the youths with TH, the youths with HL would have lower accuracy on the EU tasks. In addition, we expected that these between-group differences would be larger in the two EU paradigms that involve language comprehensions (i.e., matching emotion words with facial expressions and identifying emotions in descriptive contexts) in comparison with the other two EU paradigms (i.e., matching static facial expressions and identifying facial expressions in dynamic scenes). Second, we aimed at examining whether the parents of youths with HL and the parents of youths with TH differ in how often they express positive and negative emotions at home and whether the associations between these two aspects of parental emotional expressivity and EU are similar for the two groups of youths. We hypothesized that the parents of youths with HL would report more negative emotional expressivity compared with the parents of youths with TH. No hypothesis, however, was made for the group difference in positive emotional expressivity due to a lack of direct evidence. Moreover, we expected that the associations between parental emotional expressivity and EU would be stronger in the youths with HL in comparison with the youths with TH.

In the current study, we used human facial expressions as the response options to increase the ecological validity of the EU tasks. To guarantee the rigor of analyses, we controlled for demographic variables, including youth gender and age, as well as youths' intelligence and comprehension difficulties. All these factors have been more or less related to EU (Rieffe and Terwogt, 2006). Specifically, comprehension difficulties were included and used as a covariate because the EU tasks examined in the present study mainly differ in whether language comprehension is involved.

## Method

### Participants

Six hundred and eighty-one youths (355 boys and 326 girls) were recruited from three special education schools and two regular schools (Grades 1–9) in the southeastern area of China. We consulted the teachers at the special education schools to make sure that all the youths with HL did not have other disabilities. Because the participants were tested simultaneously in groups in the classroom, we used the following exclusion criterion to screen the participants and guarantee the quality of data: in the control task, the accuracy <100% *in conjunction with* in the four EU tasks, average accuracy <25% (the random level)^1^. Forty-nine participants (37 youths with HL and 12 youths with TH) met this criterion and were thus excluded from the analyses. These participants either were not able to understand the instructions of these tasks or just randomly responded to all the test trials without paying attention to the requirements. Compared with the youths with TH, a higher proportion of the youths with HL were excluded, $\chi_{\left(1\right)}^{2}$ = 17.43, *p* < 0.001. The excluded and included participants did not differ in the gender ratio, $\chi_{\left(1\right)}^{2}$ = 0.24, *p* = 0.12, age, *t*_(679)_ = 0.63, *p* = 0.53, maternal education status, and yearly household income, Mann—Whitney *U*-tests *Z*s < −1.80, *p*s > 0.05. The fathers of the excluded participants had a lower education level, *Z* = −2.77, *p* < 0.01.

Eventually, 632 school-aged youths were eligible for data analyses, composed of 282 youths with HL (153 boys and 129 girls) and 350 youths with TH (172 boys and 178 girls). No group difference was found in the gender ratio, $\chi_{\left(1\right)}^{2}$ = 1.63, *p* = 0.23. The youths with HL were on average 14.58 ± 3.42 years old (range: 6.97–21.80), and the youths with TH were on average 11.69 ± 2.49 years old (range: 7.47–16.56), resulting in an age gap, *t*_(630)_ = 12.36, *p* < 0.01. In China, the youths with HL are at least 2 years late when entering primary school. The hearing conditions of the youths with HL are presented in Table 1. Specifically, for the participants with TH, all of their parents had TH. For the participants with HL, two had one parent with HL, and the others had parents both with TH.

**Table 1:** Hearing conditions of youths with hearing loss (*N* = 282).

**Hearing conditions**	***n* (%)**
**Communication model**	
Spoken language	15 (5.3%)
Sign language	100 (35.5%)
Spoken and sign language	136 (48.2%)
Missing	31 (11.0%)
**Type of hearing device**	
Hearing aid	54 (19.1%)
Cochlear implant	143 (50.7%)
No device	25 (8.9%)
Missing	60 (21.3%)
**Hearing condition when born**	
No hearing loss	31 (11.0%)
Hard of hearing	56 (19.9%)
Deaf	151 (53.5%)
Unknown	24 (8.5%)
Missing	20 (7.1%)
**Hearing loss in the better ear**	
Mild (21–40 dB)	2 (0.7%)
Moderate (41–70 dB)	75 (26.6%)
Severe (71–90 dB)	109 (38.7%)
Profound (poorer than 90 dB)	50 (17.7%)
Missing	46 (16.3%)
**Hearing conditions of parents**	
Both parents have hearing loss	0 (0%)
One parent has hearing loss	2 (0.7%)
No parent has hearing loss	280 (99.3%)

Paternal and maternal education levels were lower in the youths with HL, *Z*s < −9.77, *p*s < 0.01, but the modes for both groups of parents were the high school level. Yearly household income was lower in the youths with HL, *Z* = −79.68, *p* < 0.01, and the modes were <30,000 yuan for these youths and 30,000–50,000 yuan for the youths with TH. The paternal education level, maternal education level, and yearly household income were standardized and aggregated into a socioeconomic status (SES) score with equal weight.

### Procedure and Measures

#### Emotion Understanding

EU was measured in classrooms with individual computers for ~15 min. All the participants were first asked to read the instructions of the EU tasks on the computer screen. In the instructions, the participants were informed that, if they did not understand these instructions, they could raise the hands and the experimenter (and the sign language teacher) will explain the instructions to them in person. For the youths with TH, the experimenter used spoken language to explain the instructions, whereas, for the youths with HL, the experimenter used spoken language, and the sign language teacher used sign language to explain the instructions.

A four-trial control task that simply asked participants to match the color of a dot was conducted first to help the participants understand the procedure. Four EU tasks were presented subsequently in a fixed order: a facial expression matching task, a scene matching task, a word-picture matching task, and a sentence-emotion matching task. For the convenience of the group test (e.g., the sign language teacher can identify the questions that the youths with HL might commonly ask; the experimenters can better monitor the progress of each classroom), the fixed order was used. The participants were asked to choose the correct expression from a display of four facial expressions, namely, *happiness, sadness, anger*, and *fear*. Stimuli were presented on the upper half, and these four facial expressions from the same individual were presented on the lower half of the screen. Eight individuals (four males and four females) were randomly selected from the Chinese Affective Picture System (Gong et al., 2011), and their facial expressions were used as the response options (8 × 4). Because each of the four EU tasks has eight trials, the eight individuals were randomly presented once in each EU task. The examples of the four EU tasks can be found in Supplementary Materials.

The facial expression matching task (Matsuda and Yamamoto, 2014) consisted of eight static facial expressions. Each of the four emotions was presented as the target twice. The individual in the four response options was never the same as the individual in the target picture, such that the irrelevant perceptional cues were ruled out. In the four trials, the faces of both the target and the options were from females, and, in another four trials, both were from males. The participants needed to choose the facial expression that matched the emotion expression in the stimulus.

The scene matching task (Matsuda and Yamamoto, 2014) consisted of eight 6–12-s silent clips, showing the social interactions between two performers (two males or two females). The target character interacted with another actor or actress, and one of the four emotions was elicited by those interactions. The face of the target character was masked by a mosaic, to be chosen from the four facial expressions.

The word–picture matching task (Wang et al., 2016) measured EU at a semantics-morphology level. In the eight trials, one of the four emotion words (i.e., “高兴” happiness, “悲伤” sadness, “愤怒” anger, and “害怕” fear) was served as the stimulus. The participants needed to match the emotion word with the correct facial expression.

The sentence-emotion matching task (Rieffe and Terwogt, 2006) measured EU at a semantics-syntax level. This task has eight emotion-related scenarios, and each scenario is described in a sentence. Each of the four emotions is described twice in two different sentences. The participants needed to identify the emotion described in each sentence and match it with the correct facial expression.

The reliability of the four EU tasks was good, the Cronbach's α = 0.81. The accuracy of each EU task (i.e., the proportion of correct responses) was calculated and used in our analyses. The four accuracies were significantly correlated, *r*s ranging from 0.30 to 0.43, with an average of 0.38.

#### Parental Emotional Expressivity

The participants brought home the Self-Expressiveness in the Family Questionnaire (Halberstadt et al., 1995) to their parents and were asked to bring it back completed (usually within 1 week). Either mothers or fathers were eligible for the questionnaires, and, eventually, 278 mothers (87 youths with HL and 191 youths with TH) and 200 fathers (102 youths with HL and 98 youths with TH) completed the questionnaire. This questionnaire includes a 10-item positive emotional expressivity scale, assessing the frequency of using praise and appreciation to express emotions and a 10-item negative emotional expressivity scale assessing the frequency of expressing negative emotions such as anxiety. A Likert-type scale ranging from 1 (*never*) to 9 (*very frequently*) was used. The reliability was good—for positive emotional expressivity, α = 0.85, and, for negative emotional expressivity, α = 0.81. The mean score of each scale was used.

#### Covariate: Intelligence

The Chinese version of the Raven Standard Progressive Matrices Test (Zhang and Wang, 1989) was administered through a group test for 40 min. A teacher used sign language to help the youths with HL understand the instructions and procedure of this test. Composed of six sets of matrices reason tests, the Raven test is a culturally fair assessment of reasoning and problem-solving, which does not rely extensively on linguistic knowledge. Each set has 12 items, and each item contains a matrix with one element missing, to be chosen from 6 or 8 alternatives. The reliability was excellent—α = 0.98. Since no standardized norm was available for the Chinese youths with HL, raw scores were used instead. A similar raw score was found between the youths with HL and the youths with TH, *F*_(1, 630)_ = 0.03, *p* = 0.87, possibly because of their age gap. Therefore, although the two groups of the participants differed significantly in age, they were more or less cognitively matched.

#### Covariate: Comprehension Difficulties

A battery of questionnaires was distributed to teachers who taught the Chinese language, including a measure of comprehension difficulties. Each teacher scored all the participants in the class, which usually consisted of, in the special education schools, 10–20, and, in the regular schools, around 50. Dependent on the education level, the short form of the Dyslexia Checklist for Chinese Children [DCCC (Wu et al., 2006)] was used for the children in the Grades 1–6; and the short form of the Hong Kong Specific Learning Difficulties Behavior Checklist for Junior Secondary School Students [BCL-JS (Ho et al., 2014)] was used for the adolescents in the Grades 7–9.

The 8-item meaning comprehension disorder subscale in the DCCC (e.g., “cannot understand the meaning of specific words or phrases in a given sentence”) and the 9-item Chinese reading ability subscale in the BCL-JS (e.g., “do not recognize common words”) were selected and used in this study. For both subscales, each item was rated on a Likert-type scale, ranging from 1 (*never*) to 5 (*very frequently*). The meaning comprehension disorder subscale (α = 0.95) and the Chinese reading ability subscale (α = 0.97) had good reliability. To make it possible to compare across different measures, the mean score of each subscale was standardized, separately for children and adolescents. Those standardized scores were then combined for the youths with HL and the youths with TH and analyzed. Compared with their peers with TH, the children with HL had severer comprehension difficulties, *t*_(349)_ = −5.84, *p* < 0.01. This was not found for the adolescents with HL, *t*_(279)_ = −1.51, *p* = 0.13.

## Results

The means (*M*), standard deviations (*SDs*), and correlations of all variables are presented in Table 2 separately for the youths with HL and the youths with TH. First, we conducted a two-group × 4 EU tasks ANCOVA model to examine the group differences in EU controlling for youth age and gender. The main effect of groups was significant, *F*_(12, 518)_ = 72.61, *p* < 0.001, η^2^ = 0.03, indicating that the youths with HL (estimated marginal *M* = 0.71) found the EU tasks more challenging than the youths with TH did (estimated marginal *M* = 0.79). Thus, our hypothesis was supported. Moreover, the main effect of tasks was significant, *F*_(32, 518)_ = 64.84, *p* < 0.001, η^2^ = 0.07. Using the *post-hoc* test with Bonferroni correction to probe this effect, we found that the youths had better performance on the facial expression matching task (estimated marginal *M* = 0.81) in comparison with the scene matching task (estimated marginal *M* = 0.68), *t*_(2, 518)_ = 11.19, *p* < 0.01, and the sentence-emotion matching task (estimated marginal *M* = 0.71), *t*_(2, 518)_ = 8.83, *p* < 0.01. In addition, the youths also performed better on the word-picture matching task (estimated marginal *M* = 0.80) in comparison with the scene matching task, *t*_(2, 518)_ = 10.60, *p* < 0.01, and the sentence-emotion matching task, *t*_(2, 518)_ = 8.23, *p* < 0.01. The performance did not differ between the facial expression matching task and the word-picture matching task, *t*_(2, 518)_ = 0.59, *p* = 1.00, and between the scene matching task and the sentence-emotion matching task, *t*_(2, 518)_ = −2.36, *p* = 0.11.

**Table 2:** Correlations, means (*M*), and standard deviations (*SDs*) among variables.

	**1**	**2**	**3**	**4**	**5**	**6**	**7**	**8**	***M*^a^**	***SD*^a^**	***n***
1. Positive emotional expressivity		−0.28^**^	0.01	−0.07	−0.01	−0.06	0.01	0.06	6.00	1.55	288
2. Negative emotional expressivity	−0.06		−0.09	0.12^*^	−0.04	−0.10	−0.06	−0.10	3.15	1.16	286
3. Intelligence	0.03	−0.15^*^		−0.03	0.25^**^	0.23^**^	0.21^**^	0.24^**^	31.68	22.43	350
4. Comprehension difficulties	−0.01	0.07	−0.12		0.07	0.14^*^	0.05	0.07	1.74	−0.76	341
5. Facial expression matching	−0.05	−0.17^*^	0.16^**^	−0.07		0.42^**^	0.31^**^	0.28^**^	0.84	0.20	350
6. Scene matching	0.01	−0.12	0.28^**^	−0.11	0.36^**^		0.35^**^	0.34^**^	0.67	0.22	350
7. Word-picture matching	−0.09	−0.19^*^	0.32^**^	−0.16^*^	0.45^**^	0.55^**^		0.32^**^	0.81	0.23	350
8. Sentence-emotion matching	−0.01	−0.14	0.26^**^	−0.05	0.32^**^	0.45^**^	0.46^**^		0.73	0.22	350
*M*^a^	5.71	2.96	31.94	3.53	0.79	0.69	0.80	0.69			
*SD*^a^	1.58	1.25	16.01	1.01	0.23	0.20	0.22	0.21			
*n*	189	189	282	243	282	282	282	282			

*The indexes in the upper-right area are for the youths with typical hearing; the indexes in the lower-left area are for the youths with hearing loss*.

* *p < 0.05*,

** *p < 0.01*.

a *Raw scores. Means based on estimated marginal scores after accounting for youth age and gender are reported in the main text*.

Furthermore, the interaction effect of groups × tasks was not significant, *F*_(32, 518)_ = 2.60, *p* = 0.05. The level of between-group differences was relatively similar across the facial expression matching task (estimated marginal *M*_diff_ = 0.10), the scene-matching task (estimated marginal *M*_diff_ = 0.05), the word-picture matching task (estimated marginal *M*_diff_ = 0.07), and the sentence-emotion matching task (estimated marginal *M*_diff_ = 0.10). Specifically, these mean differences between the two groups of youths calculated using the raw mean scores, and the estimated marginal scores after accounting for youth age and gender can be found in Supplementary Table 1.

To check the robustness of these results, in addition to youth age and gender, we further controlled for youth intelligence and comprehension difficulties and reran the ANCOVA model. The results were not changed. The main effect of groups, *F*_(12, 324)_ = 61.13, *p* < 0.001, and the main effect of tasks, *F*_(12, 324)_ = 63.86, *p* < 0.001, were significant. The interaction effect was still non-significant, *F*_(32, 324)_ = 2.60, *p* = 0.05^2^. We also checked whether the results were influenced by the age ranges of the two groups of participants. After weighting the age ranges and controlling for all the covariates, the main effect of groups was still significant, *F*_(12, 324)_ = 52.42, *p* < 0.001. The main effect of tasks was still significant, *F*_(32, 324)_ = 62.94, *p* < 0.001. The interaction effect was still non-significant, *F*_(32, 324)_ = 2.52, *p* = 0.06. Therefore, the results above indicated that the youths with HL found all the EU tasks more challenging, but they did not have larger differences from the youths with TH in the EU tasks that require language comprehension in comparison with the other EU tasks.

Second, we examined whether the parents of youths with HL and the parents of youths with TH differed in emotional expressivity. Before testing this research question, we had first examined whether mother-reported emotional expressivity was different from father-reported emotional expressivity. After controlling for youth age and gender, no differences were found between mothers and fathers on positive emotional expressivity, *F*_(1, 473)_ = 3.51, *p* = 0.06, and negative emotional expressivity, *F*_(1, 471)_ = 0.04, *p* = 0.84. Thus, mother-reported data were combined with father-reported data (*N* = 478; 189 parents of youths with HL, 289 parents of youths with TH, and 154 parents missing on these scales). After controlling for youth age, gender, and parent gender, no difference was found between the parents of youths with HL and the parents of youths with TH on positive emotional expressivity, *F*_(1, 472)_ = 0.004, *p* = 0.95, and negative emotional expressivity, *F*_(1, 470)_ = 2.15, *p* = 0.14. This result did not support our hypothesis that the parents of youths with HL would display more negative emotional expressivity compared with the parents of youths with TH.

Subsequently, we examined whether the associations between parental emotional expressivity and EU were similar between the youths with HL and with TH. A latent variable of EU was estimated, using the four accuracies of the EU tasks. Measurement invariance between the two groups of youths on this latent variable was estimated. We found that strict measurement invariance was tenable, $\chi_{\left(12\right)}^{2}$ = 26.36, *p* = 0.01, CFI = 0.97, RMSEA = 0.06, 90% CI [0.03,0.09], indicating that this latent variable of EU had equal factors loadings, intercepts, error variances, and latent mean structures across the two groups of youths. Based on this result, multigroup regressions were conducted (see Table 3). The model fit was good, $\chi_{\left(74\right)}^{2}$ = 123.64, *p* < 0.01, CFI = 0.92, RMSEA = 0.05, 90% CI [0.03,0.06]. Results showed that after controlling for youth age, gender, intelligence, comprehension difficulties, parent gender, and SES, negative emotional expressivity was negatively related to EU in the youths with HL, β = −0.18, *p* = 0.01, but not in the youths with TH, β = −0.09, *p* = 0.15. However, these two regression coefficients did not significantly differ: the Wald test, $\chi_{\left(1\right)}^{2}$ = 0.72, *p* = 0.40. Parental positive emotional expressivity was not related to EU in either the youths with HL, β = 0.01, *p* = 0.91, or the youths with TH, β = 0.01, *p* = 0.92.

**Table 3:** Multigroup regressions of parental emotional expressivity on emotion understanding.

	**Hearing loss**			**Typical hearing**			**Wald test**	
**Variables**	***B***	**β**	***p***	***B***	**β**	***p***	**χ^**2**^(1)**	***p***
**Covariates**								
Youth gender	−0.22	−0.09	0.15	−0.05	−0.02	0.72		
Age	0.14	0.37	0.00	0.24	0.48	0.00		
Intelligence	0.03	0.40	0.00	0.01	0.15	0.06		
Comprehension difficulties	−0.12	−0.09	0.12	−0.04	−0.02	0.72		
Parent gender	0.50	0.19	0.01	−0.08	−0.03	0.63		
SES	0.03	0.01	0.84	0.28	0.18	0.02		
**Predictors**								
Positive emotional expressivity	0.01	0.01	0.91	0.01	0.01	0.92	0.00	0.98
Negative emotional expressivity	−0.19	−0.18	0.01	−0.10	−0.09	0.15	0.72	0.40

## Discussion

Drawing from a large sample of the Chinese youths with HL and with TH and their families, we examined their group differences in EU, parental emotional expressivity, and the associations between EU and parental emotional expressivity. We found that compared with the youths with TH, the youths with HL performed less accurately on the four EU tasks. Moreover, we found that the group difference in parental emotional expressivity was non-significant, and the associations between parental emotional expressivity and EU were similarly weak for the two groups of youths.

### Group Differences in Emotion Understanding

First, the youths with HL had overall lower accuracy on the EU tasks compared with the youths with TH. This result is consistent with our hypothesis and in line with some previous studies conducted with the youths from Western cultures (e.g., Dyck and Denver, 2003; Dyck et al., 2004) and most, if not all, studies conducted with the Chinese youths (Gu et al., 2019; Wang et al., 2019). The condition of HL may affect the social learning of knowledge of emotions through at least two processes: first, youths with HL miss the auditory cues of emotion, which makes it difficult for them to integrate emotional information (Most and Aviner, 2009); and, second, because of possible communication difficulties with individuals with TH, the youths with HL miss out the opportunities to acquire emotional skills from social interactions (Netten et al., 2015). EU is an important competence, the proficiency of which is progressively achieved through participation in social interactions. The youths with HL, however, may lack sufficient participation of this type in general and thus may be likely to have delayed and, sometimes, less-optimal EU development.

Specifically, we argued that because the special education program in China is not well-designed for improving their social–emotional competence (Lytle et al., 2005), the Chinese youths with HL were at higher risk of attaining less-optimal performance on EU compared with their Chinese peers with TH. Indeed, we found that the between-group differences across the EU tasks were relatively robust. Supposedly, the youths with HL should have more social experience, consequently better EU, because they were much older than the youths with TH. Moreover, there were more youths with HL than the youths with TH, meeting the exclusion criterion who generally had very low accuracy of the EU tasks. If they were to be added to our analyses, the between-group difference in EU would have been more pronounced between the youths with HL and with TH. These participants, nevertheless, were excluded (a condition in which a significant between-group difference is less likely to happen) when the between-group difference was contrasted. Yet, even given these two situations, the Chinese youths with HL still did not catch up with their peers with TH on EU. Such a robust result may note an urgent need to reform and refine the special education system in China, which ought to at least aim at coaching and facilitating social–emotional competence simultaneously.

We did not find that, compared with the other EU tasks, the between-group differences were statistically larger in the EU tasks that involve language comprehension^3^. This finding suggests that, in spite of the developmental disadvantages related to the acquisition of language skills in the youths with HL, whether an EU task involves language comprehension did not noticeably increase the test difficulty, particularly for these youths. In another word, the role that language plays in EU development is maybe independent of or more than the “in-the-moment” language comprehension used in the EU tasks. The influence of language on EU needs to be interpreted, considering the long-term processes of development. For example, the researchers have proposed that language may help children, even infants, to learn about emotion categories in the first place through statistical learning (i.e., matching emotion concepts with emotion in one's natural environment), such that language abilities may relate to EU even without language comprehension in the tasks (Hoemann et al., 2019; Shablack et al., 2020). Indeed, our finding indicated that (a lack of) language comprehension skills explained 3% of the variance in EU, demonstrating that language is still an important factor for understanding the EU development across middle childhood and adolescence, though in the present research, its impact is not clearly evident in terms of whether language comprehension is involved in an EU task.

Similar to the youths with TH, for the youths with HL, the scene matching task and the sentence-emotion matching task were more challenging than the word-picture matching task and the facial expressions matching task. The first two tasks are mainly different from the latter two tasks in whether inferential components, rather than language comprehension components, are involved. That is, these paradigms mainly differed in whether the participants need to formulate inferences for the emotional states of the characters (Mancini et al., 2016). It has been shown that the EU tasks that involve inferential components are more challenging for children with HL, compared with those that do not (Mancini et al., 2016). Thus, in addition to the language comprehension components, future studies may consider examining whether and how the performance of the youths with HL and the youths with TH on the EU tasks varies, depending on whether the inferential components are involved.

### Group Differences in Parental Emotional Expressivity

We expected that the parents of youths with HL would display more expressions of negative emotions, because these parents have been found to be more authoritarian in their parenting compared with the parents of youths with TH (Knutson et al., 2004; Ekim and Ocakci, 2016), the parenting characteristic that has been shown to be associated with a higher level of negative emotional expressivity in the Chinese family (Chen et al., 2011). However, the results indicated that the parents of youths with HL did not differ from the parents of youths with TH in how often they express negative emotions at home. The two groups of parents also did not differ in the frequency of expressing positive emotions. Therefore, our findings tend to suggest that the possible communication difficulties and related parenting stress that the parents of youths with HL often experience did not noticeably increase their expressions of negative emotions (Knutson et al., 2004) or decrease their expressions of positive emotions. However, it should also be noted that parental emotional expressivity was measured by parents' reports, such that socially desirable responding is possible and these parents may behave distinctively in reality. Moreover, this finding needs to be interpreted in consideration of the everyday schedule of the youths. For the school-aged children and adolescents in the current study, they spent considerable time with their peers and teachers at school while relatively little time with their parents at home (excluding sleep time). As such, parent–child interactions are, maybe, not as often as they used to be when the child was at younger ages, and the parent-reported emotional expressivity was only evaluated based on their perception of these less-frequent occasions.

Furthermore, we found that negative emotional expressivity was negatively associated with EU but only for the youths with HL, and the correlation coefficients did not differ between the two groups of youths. In contrast, positive emotional expressivity was not related to EU in both groups. These results did not support our hypothesis but are congruent with the finding that the associations between parenting and empathy were similar for children with HL and with TH (Ketelaar et al., 2017). Therefore, our findings support similarities more than differences in the associations of emotion-related socialization and EU between the two groups of youths. That is, such associations are generally weak for both youths with HL and youths with TH. Such mostly non-significant correlations (especially for positive emotional expressivity) have also been shown in the meta-analytic study (Halberstadt and Eaton, 2002), questioning whether and how emotional expressivity affects EU after middle childhood.

But it is also worth mentioning that, although not significantly different from that in the youths with TH, the negative association between negative emotional expressivity and EU was significant in the youths with HL. Given that the sample size of the youths with HL was relatively smaller than the size of the youths with TH, the finding did not seem to result from the difference in the statistical power. To a certain extent, such a finding is still in line with the argument that the social–emotional development of the youths with HL depends relatively highly on parental influences (Li and Prevatt, 2010; Ketelaar et al., 2017). Parental negative emotional expressivity is different from the coaching of negative emotions (e.g., teaching emotion words) as the latter usually occurs in a calm and respectful manner. Rather, when children and adolescents are exposed to frequent expressions of negative emotions at home, they might be overaroused, which could, in turn, undermine their regulation and learning in the specific contexts (Eisenberg et al., 2003). In the Chinese family context, expressing negative emotions is not encouraged (Camras et al., 2008), as harmony is the overarching theme in Chinese families. Correspondingly, families in which high levels of negative emotions are observed could be dysfunctional, and parents often show different types of negative emotions at the same time (e.g., in an argument, sadness, anger, and frustration are expressed together). The youths from such families are less likely to refine their EU in general as they are more likely to be overaroused and less likely to take in such emotional expressions as a model for learning the knowledge of emotions. Specifically, for the youths with HL, because they could hardly pick up the auditory cues of emotional expressions, they may find it particularly difficult to decode and integrate the emotional cues when miscellaneous negative emotions are presented simultaneously. Moreover, owing to the potential communicative difficulties, these youths are also less likely to discuss with their parents about, and thus learn from, these expressions of negative emotions (e.g., causes or consequences of these emotions, and personal appraisals or physiological reactions when expressing these emotions). Eventually, they may find it relatively challenging to classify and differentiate between emotional expressions. Of course, these interpretations need to be examined in the future, using empirical data.

### Limitations and Future Directions

This study had several limitations. First, the subgroups of the youths with HL were not distinguished according to, for example, from what age they started to use hearing aids, which may have an impact on their EU (Wiefferink et al., 2012). Future studies should take these within-group variations in the youths with HL into account. Second, the age gap and the difference in the age range between the youths with HL and the youths with TH might, nevertheless, affect our results. Future studies should consider controlling for these factors (e.g., select and match participants based on both chronological ages and mental ages). Third, we used a fixed order to present the four EU tasks and the practice effect or fatigue effect is inevitable. Correspondingly, our results investigating variations between tasks with more and less requirements of language comprehension may have been influenced by the fixed order of the EU tasks as those involving language comprehension were tested last. Future research should consider using a random order between participants to handle this issue. Fourth, the missing values of parental emotional expressivity may bring about estimation biases to our results. Finally, our cross-sectional design only suggested a correlation between negative emotional expressivity and EU in the youths with HL. Using longitudinal data could further illuminate which factor precedes another one.

## Conclusions

In the current study, we examined the difference between the Chinese youths with HL and with TH in EU and found that the youths with HL have overall lower accuracies on the visual-mode EU tasks compared with their peers with TH. These findings extend the current knowledge of the social–emotional development of Chinese children and adolescents with HL. Moreover, we examined the differences between the parents of youths with HL and the parents of youths with TH in how often they express positive and negative emotions and how these aspects of parental emotional expressivity are associated with child EU. The findings showed that the two groups of parents have similar frequencies of expressing emotions, and the associations between parental emotional expressivity and EU were similar and generally weak for the two groups of youths. Therefore, these findings add to the understanding of the emotion-related socialization of the parents who have a child with HL. These conclusions provide useful information for the ongoing reform of the special education system in China, especially concerning the programs for children and adolescents with HL.

## Data Availability Statement

The raw data supporting the conclusions of this article will be made available by the authors, without undue reservation.

## Ethics Statement

The studies involving human participants were reviewed and approved by ethics committee of South China Normal University. Written informed consent to participate in this study was provided by the participants' legal guardian/next of kin.

## Author Contributions

YH and SD: substantial contributions to the conception or design of the work. FG: drafting the work or revising it critically for important intellectual content. OC: acquisition and analysis of data for the work. JC: analysis and interpretation of data for the work and final approval of the version to be published. SX: acquisition of data for the work. All authors contributed to the article and approved the submitted version.

## Conflict of Interest

The authors declare that the research was conducted in the absence of any commercial or financial relationships that could be construed as a potential conflict of interest.

## Footnotes

^1^We included four participants (3 with HL and 1 with TH) who completed the control task successfully (the accuracy = 100%) but did not achieve the random level of the average accuracy in the EU tasks (all accuracies = 12.5%) because they only met one aspect of the exclusion criterion (i.e., in the EU tasks, the average accuracy <25%). Therefore, this exclusion criterion could maximally balance the quality of data and the representativeness of participants.

^2^We also examined whether the group differences are larger in the two tasks that involve language comprehension (i.e., the word-picture matching task and the sentence-emotion matching task) in comparison with the other two tasks. In the 2 groups × 2 types of EU tasks ANCOVA model, the interaction effect was also not significant, *F*(1, 2328) = 0.73, *p* =.39.

^3^Although the interaction effect of groups × tasks could be seen as significant at a marginal level, we choose to interpret it as nonsignificant considering the relatively large sample size in our study.
