# Supplementary material for: Emotion Understanding Correlates With Parental Emotional Expressivity in Chinese Youths With Hearing Loss and Typical Hearing
Source: Front Psychol. 2021 Jun 29;12:662356. doi: 10.3389/fpsyg.2021.662356 (PMC8276695; doi:10.3389/fpsyg.2021.662356)
Supplement: Supplementary file 1 [file Data_Sheet_1.docx]

**Emotion Understanding Correlates with Parental Emotional Expressivity in Chinese Youths with Hearing Loss and Typical Hearing**

Supplementary Materials

Table S1

Raw Mean Scores and Estimated Marginal Scores for Emotion Understanding Tasks

|  | Raw Scores | | |  | Estimated Marginal Scores | | |
| --- | --- | --- | --- | --- | --- | --- | --- |
|  | Hearing Loss | Typical Hearing | *M*_diff_ |  | Hearing Loss | Typical Hearing | *M*_diff_ |
| 1. Facial expression matching | 0.78 | 0.84 | –0.05 |  | 0.76 | 0.86 | –0.10 |
| 2. Scene matching | 0.68 | 0.67 | 0.01 |  | 0.65 | 0.70 | –0.05 |
| 3. Word-picture matching | 0.80 | 0.81 | –0.02 |  | 0.77 | 0.84 | –0.07 |
| 4. Sentence-emotion matching | 0.68 | 0.72 | –0.04 |  | 0.66 | 0.75 | –0.10 |
| 5. Tasks-language not involved | 0.74 | 0.74 | 0.00 |  | 0.71 | 0.77 | –0.06 |
| 6. Tasks-language involved | 0.73 | 0.75 | –0.02 |  | 0.70 | 0.78 | –0.08 |

*Note.* Tasks that language comprehension is not involved include task 1 and task 2; tasks that language comprehension is involved include task 3 and task 4.

Estimated marginal scores are calculated after accounting for youth age and gender.

Example Items of Emotion Understanding Tasks

Figure S1


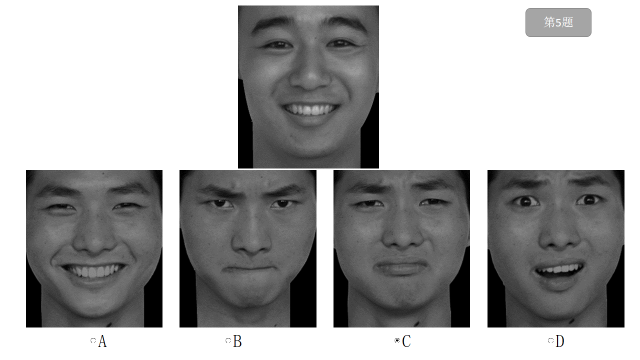
*Facial Expression Matching Task: Happiness*

Figure S2

*Scene Matching Task: Fear
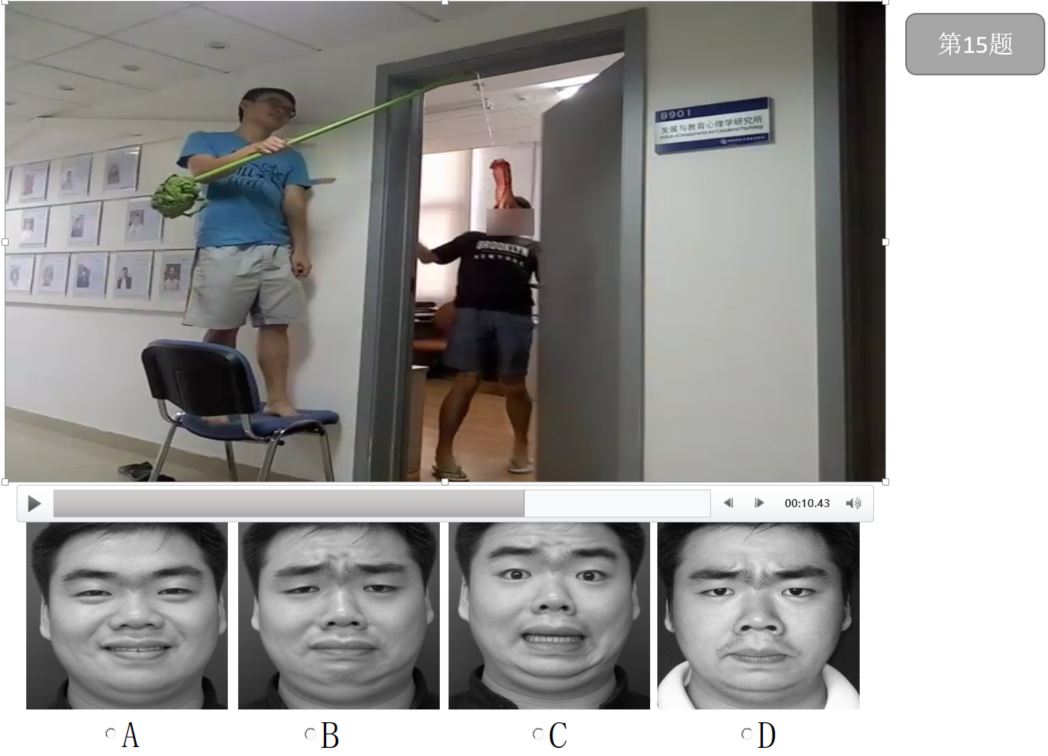
*

Figure S3

*Word-Picture Matching Task: Fear*


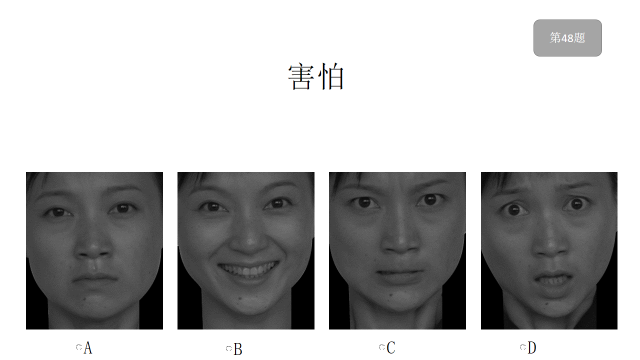


fear

Figure S4

*Sentence-Emotion Matching Task: Sad*


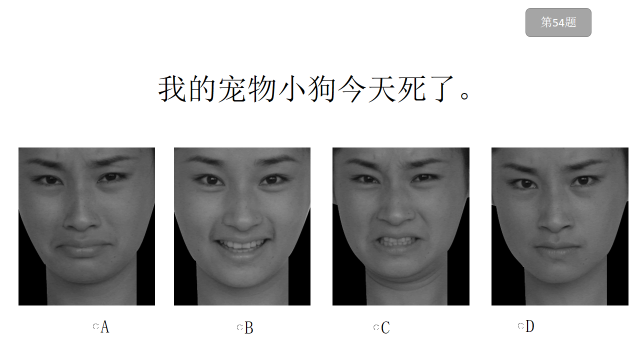


My pet puppy died today.
